# Genetic Analysis and Mapping of QTLs for Soybean Biological Nitrogen Fixation Traits Under Varied Field Conditions

**DOI:** 10.3389/fpls.2019.00075

**Published:** 2019-02-01

**Authors:** Qing Yang, Yongqing Yang, Ruineng Xu, Huiyong Lv, Hong Liao

**Affiliations:** Root Biology Center, College of Resources and Environment, Fujian Agriculture and Forestry University, Fuzhou, China

**Keywords:** QTLs (quantitative trait loci), BNF capacities, RILs (recombinant inbred lines), nodule number and nodule dry weight, nodule size, field conditions tests

## Abstract

Soybean is an important economic and green manure crop that is widely used in intercropping and rotation systems due to its high biological nitrogen fixation (BNF) capacity and the resulting reduction in N fertilization. However, the genetic mechanisms underlying soybean BNF are largely unknown. Here, two soybean parent genotypes contrasting in BNF traits and 168 F_9:11_ recombinant inbred lines (RILs) were evaluated under four conditions in the field. The parent FC1 always produced more big nodules, yet fewer nodules in total than the parent FC2 in the field. Furthermore, nodulation in FC1 was more responsive to environmental changes than that in FC2. Broad-sense heritability (*h^2^_b_*) for all BNF traits varied from 0.48 to 0.87, which suggests that variation in the observed BNF traits was primarily determined by genotype. Moreover, two new QTLs for BNF traits, *qBNF-16* and *qBNF-17*, were identified in this study. The *qBNF-16* locus was detected under all of the four tested conditions, where it explained 15.9–59.0% of phenotypic variation with LOD values of 6.31–32.5. Meanwhile *qBNF-17* explained 12.6–18.6% of observed variation with LOD values of 4.93–7.51. Genotype group analysis indicated that the FC1 genotype of *qBNF-16* primarily affected nodule size (NS), while the FC2 genotype of *qBNF-16* promoted nodule number (NN). On the other hand, the FC1 genotype of *qBNF-17* influenced NN and the FC2 genotype of *qBNF-17* impacted NS. The results on the whole suggest that these two QTLs might be valuable markers for breeding elite soybean varieties with high BNF capacities.

## Introduction

As an important economic crop, soybean is a main source of edible oil and protein for human around the world due to the high oil (20–25%) and high protein (42–45%) contents in the seeds (Aziz et al., 2016). At the same time, the high capacity of biological nitrogen fixation (BNF) found in leguminous crops, including soybean, makes this a key source of green manure in agro-ecosystems (Kumudini et al., 2008; Chen and Liao, 2017; Yang et al., 2017).

Nitrogen (N) is one of the most limiting factors in crop production. In order to obtain high yields, farmers tend to supply excessive amounts of N fertilizers, which not only increases input costs, but also causes potentially adverse effects on the environment, including air and water pollution (Santos et al., 2013; Li et al., 2016). Although N is abundant in the atmosphere, plants are unable to acquire it directly on their own, because it predominantly exists in the inert form of N_2_. BNF is a process in which plant unavailable atmospheric N_2_ is converted into readily available ammonia (NH_3_) in nodules formed through symbiotic associations between plants and microbes (Fox et al., 2016; Yang et al., 2017).

Chemical synthesis provides 118 million metric tons of fertilizer N each year (Joseph et al., 2016). In comparison, it is estimated that symbiosis between nitrogen-fixing rhizobia and plants provides 50–70 million tons of N for agricultural systems each year (Herridge et al., 2008). As one of the most important legume crops, soybean fixes 16.4 million tons of N annually, which represents 77% of the total N fixed by legume crops (Herridge et al., 2008). In modern agricultural systems, soybean is typically considered to have the most potential for sustaining green agricultural systems. For example, in Brazil, over 70% of the N required for soybean growth is derived primarily from BNF (Peoples et al., 2009). Therefore, breeding elite soybean cultivars with high BNF capacities and high yields could be an efficient way to maintain agriculture sustainability.

Soybean BNF capacity is influenced by many environmental factors. Among them, rhizobial strains play critically roles in the nitrogen fixation capacity of soybean nodules (Denison and Kiers, 2004), and which could be classed into three genotype/phenotypes: mutualistic rhizobia, parasitic rhizobia and non-symbiotic. To facilitate agriculture sustainability, mutualistic rhizobia are preferred to become parasitic and non-symbiotic rhizobia, because that parasitic and non-symbiotic rhizobia fix little nitrogen or even unable to infect legumes at all (Denison and Kiers, 2004). For example, Yang et al. (2017) found that upon inoculation with effective rhizobial strains, nodule numbers and dry weights of a RIL population did not significantly vary compared to inoculation with less effective strains, yet shoot dry weight increased by 18.25% on average. Other research has revealed that inoculation with effective rhizobium strains in soybean not only enhances nodule fresh weight, but also increases N and P contents, as well as, yield in the field (Qin et al., 2012). These results imply that rhizobial inoculation increases soybean BNF capacity mainly due to the effects of particular rhizobial species and strains. Beyond rhizobial species, BNF capacity has also been associated with soil characteristics, such as high soil nitrate levels, which tend to reduce nitrogen fixation capacity (Nohara et al., 2005; Yang et al., 2009), and low available phosphorus, which not only limits legume growth, but also inhibits BNF capacity (Adelson et al., 2000; Ward, 2011). Furthermore, water stress also appears to affect BNF capacity (Nascimento et al., 2016; Jemo et al., 2017). In short, BNF is a complex process that is affected by host and symbiotic genotypes, along with the context of environmental conditions in which the symbiosis occurs.

High BNF efficiency mainly depends on the phenotypes expressed in symbiosis between host plants and rhizobia (Nicolás et al., 2006), including the readily observable traits of nodule number (NN), nodule weight (NW), and nodule size (NS). In a relatively stable field environment, the heritability of nodulation traits may exceed 0.8 (Yang et al., 2017), suggesting that nodulation traits are mainly controlled by genetic loci. To date, a number of QTLs for nodulation traits have been identified at different stages of soybean development in pots or in field. These QTLs are distributed on several linkage groups (LGs), including D1b, A1, C2, O, B1, H, B2, E, J, and I (Tanya et al., 2005; Nicolás et al., 2006; Santos et al., 2013; Yang et al., 2017). Some QTLs, such as *qBNF-C2*, *qBNF-O* and *qBNF-B1*, are co-located with yield trait loci (Yang et al., 2017), implying that BNF capacity might affect soybean yield. Therefore, breeding new soybean varieties that produce higher yields through optimization of BNF capacity promises to be a feasible and economic strategy. Unfortunately, possibly due to technical limitations and labor costs, most of the QTLs for BNF traits obtained from field observations are preliminary and minor, so they are not likely to be useful as markers in soybean breeding programs. As a result, progress in studying BNF associated QTL markers in field experiments has lagged behind marker studies for other soybean traits.

Despite the volume of research devoted to mining QTLs for soybean BNF capacity in the field, fine mapping of QTLs regulating nodulation traits in soybean still remain largely unknown. In the present study, two soybean genotypes contrasting in nodulation traits and 168 F_9:11_ recombinant inbred lines (RILs) bred from their progeny were grown in the field to evaluate the effects of N level and rhizobial inoculation on nodulation and BNF traits, as well as, to identify associated QTLs.

## Materials and Methods

### Plant Materials and Field Trials

In this study, a soybean population consisting of 168 F_9:11_ RILs was derived from a cross between two accessions contrasting in BNF capacity, FC1 (a cultivar accession with high BNF capacity) and FC2 (a semi-wild landrace with low BNF capacity), using the single seed descent (SSD) method. This population was further used to construct a genetic linkage map to detect QTLs associated with BNF traits, as well as, to evaluate effects of N level and rhizobial inoculation on BNF traits. A field trial under four environmental conditions was carried out at the Zhaoxian experimental farm (E114.48°, N37.50°) of the Institute of Genetics and Developmental Biology, Chinese Academy of Sciences. The four environmental conditions included with (+R) or without rhizobial inoculation (-R) at two field sites contrasting in long-term nitrogen fertilization (HN: high N; LN: low N). Three highly effective rhizobium strains, BXYD3, BXBL9, and BDYD1, which were previously identified belonging to *Bradyrhizobium elkanii* based on morphological and 16s ribosomal DNA sequence analysis (Cheng et al., 2009), were used as mixture inoculants in this study. The rhizobial inoculation treatment was applied to seeds as described by Qin et al. (2012). Briefly, soybean seeds were uniformly mixed with rhizobium inoculants before planting. The soil at the experiment site is a *Fluventic Ustochrept* soil. Basic characteristics of the top 20 cm of soil in the HN and LN field are shown in Table 1. The previous crop was wheat, which was fertilized with 202.5 kg⋅ha^-1^ of (NH_4_)_2_HPO_4_ and 187.5 kg⋅ha^-1^ of urea as base fertilizer, and 75 kg⋅ha^-1^ of urea as additional fertilizer during the elongation stage in the HN field. No fertilizer was applied during soybean growth. Field management (i.e., pest control and irrigation, etc.) followed local farmer practices. Parental genotypes and RILs were planted in a split plot design with plots arranged in randomized complete blocks within each block of split plots. There were two plots for each RILs in each treatment, and in total, were 1,360 plots in the field. Thirty seeds were sown per plot in three 3 m rows spaced 0.5 m apart.

**Table 1:** Basic characteristics of the field soils where the tested soybean lines were grown.

Field	pH	Nitrate N (mg⋅kg^-1^)	Available P (mg⋅kg^-1^)	Available K (mg⋅kg^-1^)	Organic matter (g⋅kg^-1^)
HN	8.12 ± 0.10	90.25 ± 9.51	16.17 ± 2.96	89.53 ± 12.53	1.93 ± 0.20
LN	8.04 ± 0.08	58.17 ± 7.10	14.70 ± 2.24	87.75 ± 8.50	1.97 ± 0.27

### Plant Sampling and Measurements

At the R4 stage, three representative plants from each plot were harvested, and in total, six plants were harvested for each line in each treatment. Plant roots were manually extracted from the soil to ensure the integrity of the root systems. All nodules were removed and cleared carefully from roots prior to drying in a 37°C oven. Three nodulation traits, namely NN, NW, and NS, were investigated for subsequent analysis. NS was calculated by the quotient of total NW and NN. NN and NW were derived from individual plant. The plants were dried at 60°C, and then measured dry weight and total N content (TNC) using a continuous flow analyzer as described by Bao (2000). The TNC was used to represent the BNF capacity.

### Genotyping by High-Throughput Sequencing

Genomic DNA of the two parents were extracted using commercial extraction kit, DNeasy Plant Mini Kit (QIAGEN, Germany), following the manufacturer protocol. Library construction and sequencing were performed on the Illumina sequencing platform. Briefly, genomic DNA was fragmented to 100–300 bp size and two replicate genomic DNA re-sequencing libraries (100–300 bp insert) were prepared for two entire lanes sequencing on Hiseq X10 PE150 sequencing system. Genotyping by sequencing (GBS) libraries were constructed using the EcoRI and NIaIII enzymes modified from the Elshire’s protocol (Elshire et al., 2011). The simple description as follows: after genomic DNA extracting, 100 ng of DNA for each plant were used for digesting reaction with EcorI and NIaIII (New England Biolabs, Ipswich, MA, United States) in 96-well plates. Then, the reactions were mixed with about 25 pmol of unique A1 and A2 adapters per well. The libraries in 96-well plates were then pooled, further DNA fragments size 400–600 bp were isolated on a 1% agarose gel and purified using a PCR purification kit (NEB), and then amplified for 12 cycles using Phusion DNA polymerase (NEB). Finally, the pooled libraries were adjusted to 10 nmol and sequenced with PE125 on the HiSeq4000 (Illumina, San Diego, CA, United States). Both genomic DNA re-sequencing and GBS were performed by Genedenovo Biotechnology Co., Ltd. (Guangzhou, China).

### SNP Identification and Bin Map Construction

For SNPs calling, the Burrows-Wheeler Aligner (BWA) was used to align the clean reads from each plant against the reference genome *Glycine max* Wm82.a2.v1 with the settings ‘mem 4 -k 32 -M’ (Li and Durbin, 2009). Genotypes of all samples were determined by using the GATK’s Unified Genotyper. SNPs were filtered using GATK’s Variant Filtration with follow parameters (-Window 4, -filter “QD < 2.0 ||FS > 60.0|| MQ < 40.0,” -G_filter “GQ < 20”) and SNPs with segregation distortion or sequencing errors were eliminated. The physical positions of each SNP were further determined by using the software tool ANNOVAR (Wang et al., 2010). Polymorphic parental markers were recorded as male genotype “a” and female genotype “b,” other segregation patterns were recorded as missing data “-.” However, the reading depth of SNP variants outside the range of 5–1,500 would be also considered as missing data. After that, Chi-square (χ^2^) tests were conducted for all SNPs to detect segregation distortion. The sliding window (1 kb) approach was used to evaluate groups of consecutive SNPs for utility in genotyping and avoidance of false positive population based SNPs. Qualified bins of SNP markers were then used to construct genetic linkage maps using Join Map 4.1. The regression algorithm and Kosambi mapping function were used in marker distance calculation. The LOD value was set according to the chromosome numbers. A Perl SVG module was used to draw the linkage map. Bin markers were named as Ch“x”.“y” according to results of SNP calling, “x” and “y” represent “chromosome number” and “physical position,” respectively.

### QTL Detection

Multiple-QTL model (MQM) implemented in MapQTL6.0 (Van Ooijen, 2009) was used to detect QTLs for nodulation and BNF capacity traits. The critical logarithm of odds (LOD) threshold was set to 4.5 for declaring the significance of a QTL in a particular genomic region. A total of 1,000 permutations with *P*-values <0.05 was used to verify LOD scores. QTLs for which the LOD score simultaneously exceeded 4.5 and the genome wide LOD, were considered as high confidence QTLs in MQM mapping.

### Statistical Analysis

Data for the three BNF traits were used for variance and QTL analysis. Analysis of variance (ANOVA) was implemented in the QTL ICIMapping V4.1 software (Meng et al., 2015). Parental genotypes were also planted with six replications, which were analyzed separately from the RILs. Broad sense heritability (*h^2^_b_*) was estimated for each trait according to: *h^2^_b_* = VG/(VG + VE), with VG as the variance between RILs and VE as the variance within RILs. The Student’s *t*-test was used to test the significance of the effects of N level and rhizobial inoculation on each trait in RILs. Multi-factor and multivariate analysis of variance (ANOVA) was also performed using SPSS 19 (Colin and Paul, 2012).

## Results

### Phenotype Evaluation of Two Parents

**Figure 1:**
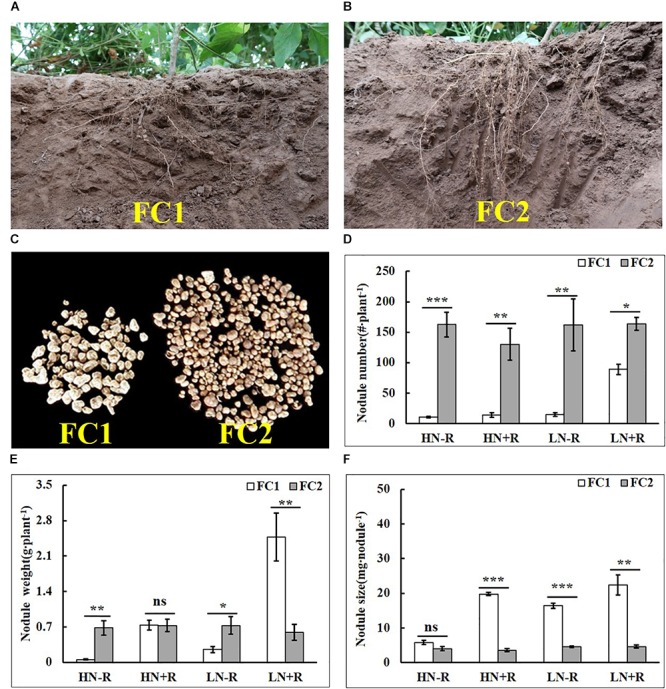
Root and nodule characteristics for FC1 and FC2 soybean parental lines in the field under four environment conditions. **(A,B)** Root and nodule characteristics of FC1 and FC2. **(C)** Nodule size and number for FC1 and FC2 under natural conditions. **(D–F)** Nodule number, nodule weight, and nodule size on roots of FC1 and FC2 under four environments. Bars represented means ± SE from six replications. Asterisks indicated significant differences between FC1 and FC2 in the Student *t*-test at 5% (^∗^), 1% (^∗∗^) and 1‰ (^∗∗∗^) levels, while “ns” represented no significant difference. “HN” and “LN” denoted high and low nitrogen, “+R” and “-R” denoted the inoculation or non-inoculation of rhizobium.

The two parental genotypes, FC1 and FC2, contrasted in root growth and nodulation in the field under natural conditions. ANOVA revealed significant genotypic variation between parental genotypes for NN (*P* < 0.001) and NS (*P* < 0.01), as well as, a significant interaction between rhizobium inoculation and genotype on NW (*P* < 0.05) (Table 2). Compared with FC2, FC1 had a shallower root architecture denoted by more roots in the top soil, and more big nodules than FC2 in the field (Figures 1A–C). However, FC2 formed more nodules than FC1 in each environment, as indicated by 83.90–1473.09% differences in NN under the four tested environments (Figure 1D). Interestingly, NW of FC2 was only higher than that of FC1 in plots without rhizobial inoculation (-R), but not in HN+R or LN+R (Figure 1E). Nodules on FC1 roots were much bigger than on FC2 roots in all treatments except HN-R, with NS being 450.11%, 260.89%, and 382.50% bigger for FC1 compared to FC2 in HN+R, LN-R, and LN+R, respectively (Figure 1F), indicating that FC1 was more conducive to nodule development in response to rhizobial inoculation.

**Table 2:** ANOVA for variation in biological nitrogen fixation traits between two parental genotypes.

Traits	R	N	G	R × N	R × G	N^∗^G	R × N × G
NN	0.54	0.53	5.03E-06^∗^	0.36	0.13	0.13	0.87
NW	0.22	0.26	0.94	0.21	0.02^∗^	0.12	0.52
NS	0.17	0.88	0.01^∗^	0.71	0.13	0.99	0.6

*R, rhizobium inoculation; N, nitrogen level; G, genotype; R **×** N, interaction effects of rhizobium inoculation and nitrogen level; R **×** G, interaction effects of rhizobium inoculation and genotype; N **×** G, interaction effects of nitrogen level and genotype; R **×** N **×** G, interaction effects of rhizobium inoculation, nitrogen level and genotype. Asterisks indicated significant differences through ANOVA at the 5% (^∗^) level*.

In addition, N level significantly influenced nodulation on FC1 but not FC2. NN increasing by 535.71% or 41.94%, NW increasing by 323.70% or 235.96%, and NS increasing by 12.90% and 181.63% in response to rhizobium inoculation in LN and HN treatments, respectively (Figures 1D–F). These results strongly suggest that nodulation traits are much more sensitive to environment changes for FC1 than for FC2, which might be controlled by genetic loci.

### Phenotypic Variation Among Recombinant Inbred Lines (RILs)

**Figure 2:**
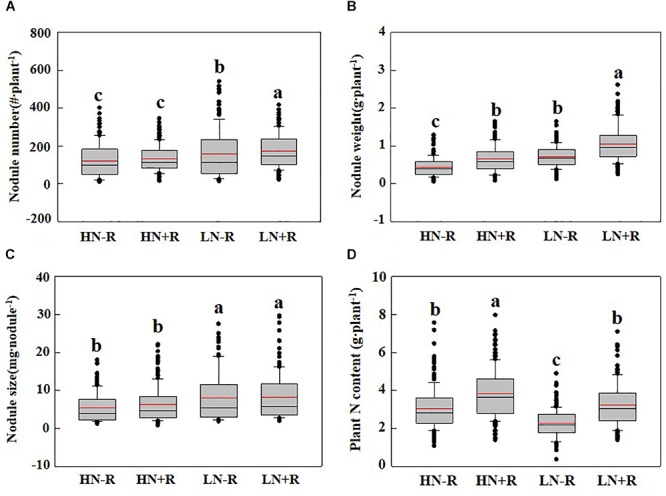
Biological nitrogen fixation (BNF) traits as affected by N level and rhizobium in RILs. **(A)** Nodule number; **(B)** nodule weight; **(C)** nodule size; and **(D)** plant nitrogen content. The black and red lines, lower and upper edges, and bars above or below the boxes represented median and mean values, 25th, 75th, 5th, and 95th percentiles of all data, respectively. Different letter(s) over error bars indicated significance differences between HN-R, HN+R, LN-R, and LN+R conditions through the Student *t*-test at the 1% level.

As shown in Table 3, significant phenotypic variation existed for all three nodulation traits within the 168 F_9:11_ soybean RILs under different environments. As expected, among the RILs, high N supply significantly suppressed nodule formation and development as indicated by declines of 33.31% and 30.91% in NN, 63.72% and 63.72% in NW, and 29.87% and 51.98% in NS, with or without rhizobial inoculation, respectively (Figures 2A–C). Furthermore, TNC increased in response to rhizobial inoculation at both N levels, with a greater impact in LN than in HN (Figure 2D), suggesting that BNF capacity is inhibited by high N supply in the RIL population.

**Table 3:** Phenotypic variation and genetic analysis of biological nitrogen fixation traits using 168 F_9:11_ soybean RILs under different field conditions.

Trait	Environment	Parents		RILs						
		FC1	FC2	Mean ± SD	Min	Max	CV%	*h^2^_b_*	Skew	Kurt
NN (#⋅plant^-1^)	HN-R	10.33 ± 3.01	162.5 ± 49.49	119.45 ± 129.22	1	985	108.18	0.76	0.76	-0.05
	HN+R	14 ± 9.64	130.2 ± 65.08	131.10 ± 116.15	1	938	88.60	0.48	0.88	0.26
	LN-R	14.67 ± 6.66	162 ± 103.68	156.36 ± 164.38	1	1047	105.13	0.83	1.02	0.31
	LN+R	89 ± 21	163.67 ± 26.86	174.78 ± 130.44	4	835	74.63	0.63	0.65	-0.33
NW (g⋅plant^-1^)	HN-R	0.06 ± 0.02	0.68 ± 0.35	0.43 ± 0.40	0	2.51	91.96	0.62	1.01	1.03
	HN+R	0.74 ± 0.24	0.73 ± 0.31	0.66 ± 0.57	0	5.78	85.96	0.69	0.78	0.15
	LN-R	0.25 ± 0.15	0.73 ± 0.42	0.72 ± 0.50	0	3.56	70.08	0.67	0.49	0.12
	LN+R	2.48 ± 1.16	0.6 ± 0.39	1.06 ± 0.70	0	4.16	66.54	0.70	1.12	1.45
NS (mg⋅nodule^-1^)	HN-R	5.83 ± 1.57	4.01 ± 1.4	6.36 ± 6.30	0.16	53.8	99.03	0.70	1.57	2.99
	HN+R	36.77 ± 29.41	3.6 ± 1.17	7.12 ± 7.13	0.2	87.38	100.2	0.75	1.38	1.37
	LN-R	16.42 ± 1.77	4.55 ± 0.63	9.93 ± 9.77	0.26	81.21	98.34	0.87	1.64	2.98
	LN+R	22.35 ± 7.1	4.63 ± 1.19	8.81 ± 7.86	0	73.15	89.19	0.86	1.36	1.81

*RILs, recombinant inbred lines; NN (nodule number, #⋅plant^-*1*^); NW (nodule weight, g⋅plant^-*1*^); NS (nodule size, mg⋅ nodule^-*1*^); HN (high nitrogen condition); LN (low nitrogen condition); -R (without rhizobium inoculation); +R (with rhizobium inoculation); Min and Max, the minimum and maximum values of NN, NW, and NS; CV%, coefficient of variation; *h^*2*^_*b*_*, broad-sense heritability; Skew and Kurt, Skewness and Kurtosis.*

The mean value for each trait among all RILs was between the mean values of the two parents, while the maximum and minimum values were beyond the extremes of the two parents. Distributions for the nodulation traits under all conditions were approximately normal according to Kurtosis and Skewness values calculated over six replicates. Broad-sense heritability (*h^2^_b_*) for all the traits under the tested environments varied from 0.48 to 0.87, with generally higher values being observed in LN than in HN plots (Table 3). Overall, the results herein suggested that variations in nodulation traits mainly depended on genotype.

### Construction of Genetic Linkage Map

After strict screening, 168 strains of the RIL population were accepted and genotyped using a sliding-window method (Chang and Lee, 2004). Bin markers for each chromosome were screened, and a total of 3,319 recombinant bin markers were identified (Table 4). A genetic map was constructed by mapping these 3,319 bin markers onto the 20 soybean chromosomes (Supplementary Figure S1). The complete genetic distance of the linkage map was 2,537.6 cM. The average distance between two adjacent markers was 0.76 cM. Among the 20 soybean chromosomes, chromosome 7 had the highest number of bin markers at 194, which cover a genetic length of 157.9 cM. Chromosome 10 held the fewest markers, 100, over a distance of 101.0 cM. The *W* gene located on chromosome 13 has been identified in previous work as responsible for flower color (Takahashi et al., 2010). In order to examine the fidelity of the genetic map constructed herein, this flower color gene was mapped relative to the bin markers used in the current study. As expected, the gene encoding flower color was mapped as the *W* gene located on chromosome 13 at bin marker Chr13.17168452, with a high LOD value of 74.56 (Supplementary Figure S1). This clearly demonstrated that the linkage map constructed in this study is a high quality map that can be used in further studies.

**Table 4:** Summary of bin marker characteristics of recombinant inbred line (RIL) population.

Chr	Genetic distance/cM	Physical length	Number of bin markers	Average coverage distance/cM
Chr01	123.1	56510449	169	0.73
Chr02	131.0	47706575	165	0.79
Chr03	140.0	45730193	169	0.83
Chr04	158.1	51405255	188	0.84
Chr05	97.5	41919006	140	0.70
Chr06	152.2	50892625	177	0.86
Chr07	157.9	43534570	194	0.81
Chr08	186.5	47709553	174	1.07
Chr09	155.5	50173153	131	1.19
Chr10	101.0	51542279	100	1.01
Chr11	168.5	34671664	110	1.53
Chr12	119.4	40030102	178	0.67
Chr13	121.8	45514498	175	0.70
Chr14	83.7	47841279	171	0.49
Chr15	75.0	51710377	191	0.39
Chr16	76.1	37192783	178	0.43
Chr17	137.0	41348852	178	0.77
Chr18	103.2	57961359	182	0.57
Chr19	82.3	50522110	175	0.47
Chr20	167.7	47868276	174	0.96
Total	2537.6	941784958	3319	0.76

### QTL Identification for Nodulation Traits

A total of 12 significant QTLs explaining 12.6–59% of the phenotypic variation observed among the 168 F_9:11_ soybean RILs were identified for nodulation traits in the field under four environment conditions (Table 5). The LOD values of these QTL varied from 4.93 to 32.52. However, 10 of the QTLs were closely mapped on Chromosome 16, and the other two QTLs were mapped on Chromosome 17 at two very closely linked locations. These results indicating that all of the significant QTLs might be controlled by two genetic loci, which were, therefore, named as *qBNF-16* and *qBNF-17*. Among them, *qBNF-16* is a stable and major QTL, which was detected under all four of the environment conditions, and explained 40.9%–59% and 15.9%–45.4% of the variation in NS and NN, respectively. The additive effects of *qBNF-16* for NS and NN were derived from FC1 and FC2, respectively. In contrast, *qBNF-17* was only detected in LN-R, and it explained 18.6% and 12.6% of the variation in NN and NW, respectively. The additive effects of *qBNF-17* for NN and NW were derived from FC1 and FC2, respectively. As for interactions between traits, the results in this study indicated that NN is negatively correlated with NS.

**Table 5:** Putative QTLs detected for biological nitrogen fixation traits using 168 F_9:11_ soybean RILs in the field.

Integrated QTL^a^	Environment^b^	Separate QTL	Chr	Marker or interval^c^	Position (cM)	LOD	Add^d^	PVE (%)^e^
*qBNF-16*	HN-R	*qNN-HN-R*	Gm16	Chr16.37147484	17.63	22.12	65.49	45.4
		*qNS-HN-R*	Gm16	Chr16.37147484	17.63	25.25	-4.78	50.0
	HN+R	*qNN- HN+R*	Gm16	Chr16.37559118	18.55	6.31	29.19	15.9
		*qNW-HN+R*	Gm16	Chr16.36986426	16.09	7.60	-0.17	18.8
		*qNS*-*HN+R*	Gm16	Chr16.37147484	17.63	19.18	-3.31	40.9
	LN-R	*qNN-LN-R*	Gm16	Chr16.37156866	17.87	15.63	69.79	28.4
		*qNS-LN-R*	Gm16	Chr16.37147484	17.63	30.50	-6.01	56.7
	LN+R	*qNN-LN+R*	Gm16	Chr16.37147484	17.63	14.91	55.68	33.6
		*qNW-LN+R*	Gm16	Chr16.36986426	16.09	10.12	-0.25	24.2
		*qNS-LN+R*	Gm16	Chr16.37147484	17.63	32.52	-5.08	59.0
*qBNF-17*	LN-R	*qNN-LN-R*	Gm17	Chr17.12638388	61.78	7.51	-55.58	18.6
		*qNW-LN-R*	Gm17	Chr17.12728155- Chr17.13805621	60.24	4.93	0.40	12.6

^a^*BNF, biological N_*2*_ fixation. ^*b*^LN and HN mean low and high N fertilizer field, respectively, and “-R” and “+R” mean without and with rhizobial inoculation, respectively. ^*c*^Marker or interval, markers or support intervals on the linkage map in which the LOD is the largest. ^*d*^Add value >0 and <0 stand for increasing effects of the QTLs derived from FC2 and FC1, respectively. ^*e*^PVE (%), percentage of phenotypic variance explained by the QTL*.

### Effects of Genotype on Nodulation Traits Under Varied Environmental Conditions

Biological nitrogen fixation capacity can be represented by plant total nitrogen content (TNC) (Li et al., 2016). In order to evaluate the effects of *qBNF-16* and *qBNF-17* on nodulation traits, the 168 F_9:11_ lines were classed into two distinct genotypic groups based on bin markers Chr16.37147484 and Chr17.12638388. Then, comparative analysis of nodulation traits and TNC were conducted among different environments between these two groups.

**Figure 3:**
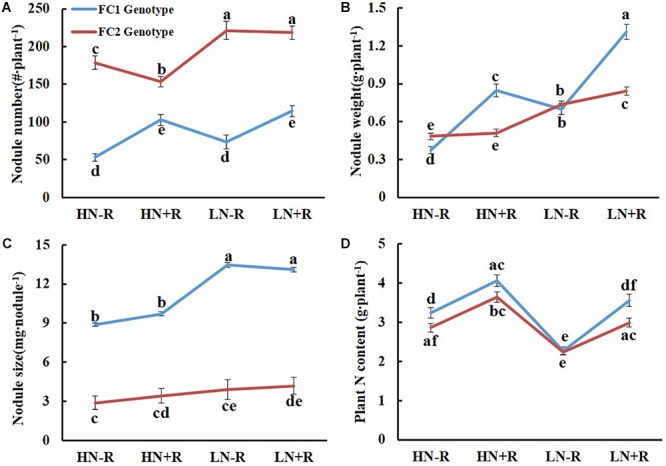
Effects of qBNF-16 genotypes on BNF traits. **(A)** Nodule number; **(B)** nodule weight; **(C)** nodule size; and **(D)** whole plant nitrogen content. Based on SNP marker Chr16.37147484, the RIL population was divided into FC1 and FC2 genotype groups which consisted of 72 and 92 lines, respectively. The blue and red line represented mean ± SE values for the FC1 and FC2 genotype groups. Different letter(s) over the standard error bar indicated significant differences of mean values at the 5% level.

The genotype of *qBNF-16* significantly affected NN and NS, but not NW and TNC (Figure 3). The FC1 group had fewer, yet larger nodules than the FC2 group (Figures 3A,C). This suggested that the FC1 genotype of *qBNF-16* supported nodule development, while the FC2 genotype of *qBNF-16* promoted nodule formation. Furthermore, the FC1 genotype of *qBNF-16* was responsive to rhizobial inoculation, as indicated by increases of NN of 93.20% and 55.34%, NW of 126.97% and 87.08%, and TNC of 25.18% and 56.56% upon rhizobial inoculation in HN and LN plots, respectively. Meanwhile, NS was only affected by N level. The FC2 genotype of *qBNF-16* was also influenced by N level, as showed by decreases in NN of 42.04% or 24.07%, and in NW of 64.69% or 51.69% in HN plots with or without rhizobial inoculation, respectively.

**Figure 4:**
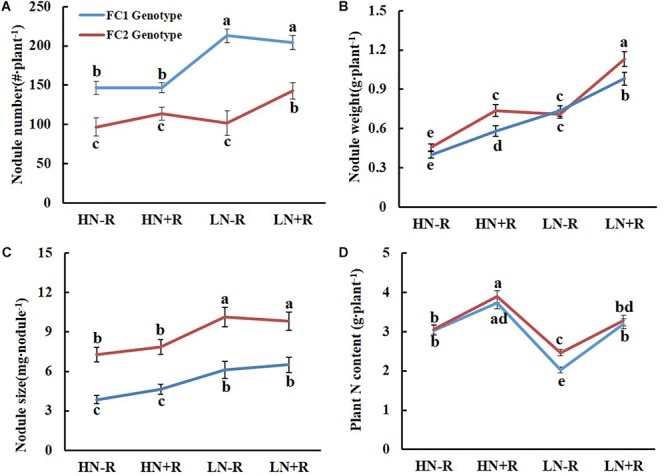
Effect of qBNF-17 genotypes on nodulation traits. **(A)** Nodule number; **(B)** nodule weight; **(C)** nodule size; and **(D)** whole plant nitrogen content. Based on SNP marker Chr17.12638388, the RIL population was divided into FC1 and FC2 genotype groups which consisted of 83 and 81 lines, respectively. The blue and red line represented mean ± SE values for the FC1 and FC2 genotype groups. Different letter(s) over the standard error bar indicated significant differences of mean values at the 5% level.

The genotype of *qBNF-17* also only significantly affected NN and NS, but had opposite effects compared to *qBNF-16* (Figure 4) impacts. The FC2 group had fewer, but bigger nodules than the FC1 group (Figures 4A,C), suggesting that the FC2 genotype of *qBNF-17* participated in nodule development, while the FC1 genotype of *qBNF-17* advanced nodule formation. Both N level and rhizobial inoculation significantly impacted NW and TNC for both FC1 and FC2 genotypes of *qBNF-17*, while only N level affected NS, and rhizobial inoculation only influenced NN for the FC2 genotype at the *qBNF-17* locus in LN plots.

## Discussion

By 2050, agricultural production might need to be increased by 70% in order to satisfy the needs of a growing population (Joseph et al., 2016). In order to achieve higher yields, farmers tend to supply excessive amounts of fertilizers, which often leads to environmental problems (Li et al., 2016). Legumes, particularly soybean, not only could provide oil and protein for humans, but also can improve soil quality and reduce the need for N fertilizers. This benefit of growing legumes is due to the unique capability among this family to participate in BNF, which converts atmospheric nitrogen (N_2_) into ammonia with the assistance of rhizobia in symbiotically derived organs known as nodules (Udvardi and Poole, 2013). Identification and fine mapping QTLs for BNF capacity hold the promise of facilitating the development of soybean cultivars with higher yield potentials and superior BNF capacities that will improve our ability to meet food and environment demands.

Soybean yield has been demonstrated being largely dependent on root architecture (RA) and BNF capacity (Li et al., 2016; Chen and Liao, 2017; Yang et al., 2017), and the effects of RA and BNF capacity on yield are also associated with each other. For example, RA of legume roots is not only involved in elaboration of root system through lateral root formation, but also in nodule formation with rhizobia from soils (Yendrek et al., 2010). However, information of the relationships between RA and BNF remains limited. Our recent research suggested that the soybean plants with shallow RA had more and heavier nodules, as well as higher SDW. Meanwhile, soybean roots became shallower after inoculating with rhizobia, showing strong synergistic interactions between RA and BNF (Yang et al., 2017). In this study, the two soybean accession FC1 and FC2 contrasting in RA were evaluated in varied field conditions. Interestingly, we found that the parental genotype FC1 with shallow RA preferred to form fewer but bigger nodules compared with the parental genotype FC2 with deep RA, implying that more complicate relationships between RA and BNF exited and need to be further evaluated.

Since effective rhizobial strains play critical roles in facilitating soybean production with high nitrogen fixation capacity, lots of commercial rhizobial strains have been isolated and applied in the field (Menna et al., 2006; Thuita et al., 2012; Sivparsad et al., 2015). However, none of the commercial rhizobial strains could meet the needs for all crop species and/or all cultivars in the same species in the field (Thomas-Oates et al., 2003). In this study, FC2 had a higher nodulation ability with numerous small nodules than FC1, which was possibly caused by non-selectivity compatibility of FC2 to most indigenous rhizobia, and this non-selectivity compatibility greatly related to plant species (i.e., genotype). On the other hand, phenotypes of both FC1 and FC2 were the results of synergetic interactions between indigenous rhizobia (most likely including parasitic rhizobia), inoculants, genotype and other environmental factors. However, the BNF capacity of small nodules (diameter <2 mm) was significantly lower than that of big nodules (diameter >2 mm) (Li et al., 2018). Contrastingly, FC1 seemed to have a higher compatibility with the inoculated rhizobial strains than indigenous soil rhizobia as indicated by great increases of NN and NW after rhizobium inoculation, especially at low N level (Figure 1). Since highly effective and affinitive rhizobial species are necessary for high BNF capacities to produce high yields in soybean (Peoples et al., 2009; Qin et al., 2012; Yang et al., 2017), TNC was measured for the RIL population to evaluate the effect of a highly effective and affinitive rhizobial species on soybean BNF capacity in the field. As expected, inoculation with highly effective and affinitive rhizobia led to 44.5% and 25.1% increase of TNC of RILs in low and high N conditions, respectively (Figure 2). Furthermore, the mean value of TNC in RILs did not significantly vary between HN-R and LN+R treatments, suggesting that rhizobium inoculation is an alternative way to meet N demand for soybean growth.

Nodulation traits that heavily influence BNF capacity are affected by many environment factors, such as nutrient availability, soil characteristics, abiotic stress, and soil tillage systems (Soedarjo et al., 2003; Adak and Kibritci, 2016; Chetan et al., 2016; Kunert et al., 2016). In soybean, although symbiotic N_2_ fixation can provide the nitrogen needed for plant growth, symbiotically fixed N is not always sufficient for producing high yields (Kunert et al., 2016). High N fertilizer application on the other hand has a negative impact on nodulation (Ferguson et al., 2010). In this study, BNF traits was studied in a field with low N and high N plots in which variation in nodule formation could proceed with or without rhizobial inoculation. As expected, the mean values of three BNF traits (i.e., NN, NW and NS) were significantly higher by 32.2–62.3% in low N plots than in high N plots, indicating that BNF capacities are higher when N is limited (Table 3). This is consistent with previous reports that high N status has a negative impact on nodulation traits (Ferguson et al., 2010; Abera and Tadele, 2016; Adak and Kibritci, 2016; Hu et al., 2016; Xia et al., 2017).

Genetics are the internal elements that might affect soybean BNF capacity along with environmental elements. Therefore, identification and mapping of QTLs for BNF traits holds promise for breeding efforts seeking to develop soybean varieties with high BNF capacities, which will allow growers to reduce N fertilization and maintain beneficial eco-systems. To successfully reach these goals, a high *h^2^_b_* is necessary for identified solid QTLs. In this study, the *h^2^_b_* for NW and NS are stable under different environments, with values ranging from 0.62 to 0.70 and 0.70 to 0.87, respectively. For NN, rhizobial inoculation significantly decreased values of *h^2^_b_* to 0.48 and 0.63 in HN and LN field plots (Table 3), respectively, while other *h^2^_b_* values for nodulation traits remain above 0.75 without rhizobial inoculation under both LN and HN field conditions. This high level of heritability is consistent with previous reports (Souza et al., 2000; Santos et al., 2013; Yang et al., 2017). This strongly indicates that genetics is the main force affecting nodulation trait variation in the field, and, furthermore, the RIL population developed in this work is suitable for QTL identification.

Nodulation traits are difficult to quantify and study in field conditions. Although more than 20 QTLs for BNF traits have been identified in previous studies^1^, most of them were not adequately tested in field conditions, or there is a lack of adequate repetition required for obtaining precise QTL effect estimates. In addition, a high resolution genetic map is also necessary for precise QTL identification. Unfortunately, none of previous QTLs reports built on SSR based genetic maps exhibit adequate precision, possibly due to technical limitations. Therefore, it is not surprising that among previously identified QTLs for nodulation traits, relatively low LOD values ranging from 1.28 to 8.77 were detected, and none of them could explain more than 40% of variation (López-Bucio et al., 2003; Tanya et al., 2005; Nicolás et al., 2006; Santos et al., 2013; Yang et al., 2017). In this study, QTLs were precisely identified by using six representative plants for phenotypic observations, while a high resolution genetic map was constructed to detect QTLs under multiple environments. As a result of this effort, a QTL, *qBNF-16*, explaining as much as 59% of genetic variation, and with LOD values of up to 32.52 was identified and mapped. Most importantly, *qBNF-16* is a stable QTL that was detected under all four tested environments. Therefore, *qBNF-16* can be considered as a valuable locus which might be useful for breeding high BNF capacity soybean varieties.

Integration of QTLs for BNF traits in a previous reported reveals that a total of 18 QTLs affect BNF traits under field conditions (see footnote 1). These QTLs are distributed on linkage groups D1a(2), C1(1), A1(1), C2(4), A2(1), K(1), B1(1), E(2), L(2), and I(3). None of these QTLs co-locate or are closely linked with QTLs identified in our study, suggesting that *qBNF-16* and *qBNF-17* are two new QTLs. Taken together, in this study, two new QTLs for soybean nodulation and BNF capacity traits in the field conditions were identified and tested under different environments. The results indicate that these QTLs can be used as molecular markers for breeding elite soybean varieties with high BNF capacities.

## Data Availability Statement

Any related SNPs data can be available on request by contacting the corresponding author directly.

## Author Contributions

HLi designed the experiments and critically revised the manuscript. QY and YY analyzed the data and wrote the manuscript. QY, YY, RX, and HLv carried out the experiments.

## Conflict of Interest Statement

The authors declare that the research was conducted in the absence of any commercial or financial relationships that could be construed as a potential conflict of interest.

## Footnotes

1 https://www.soybase.org/
