# Supplementary material for: Genetic Analysis and Mapping of QTLs for Soybean Biological Nitrogen Fixation Traits Under Varied Field Conditions
Source: Front Plant Sci. 2019 Feb 1;10:75. doi: 10.3389/fpls.2019.00075 (PMC6367678; doi:10.3389/fpls.2019.00075)
Supplement: Supplementary file 1 [file Presentation_1.PPTX]

## Slide 1
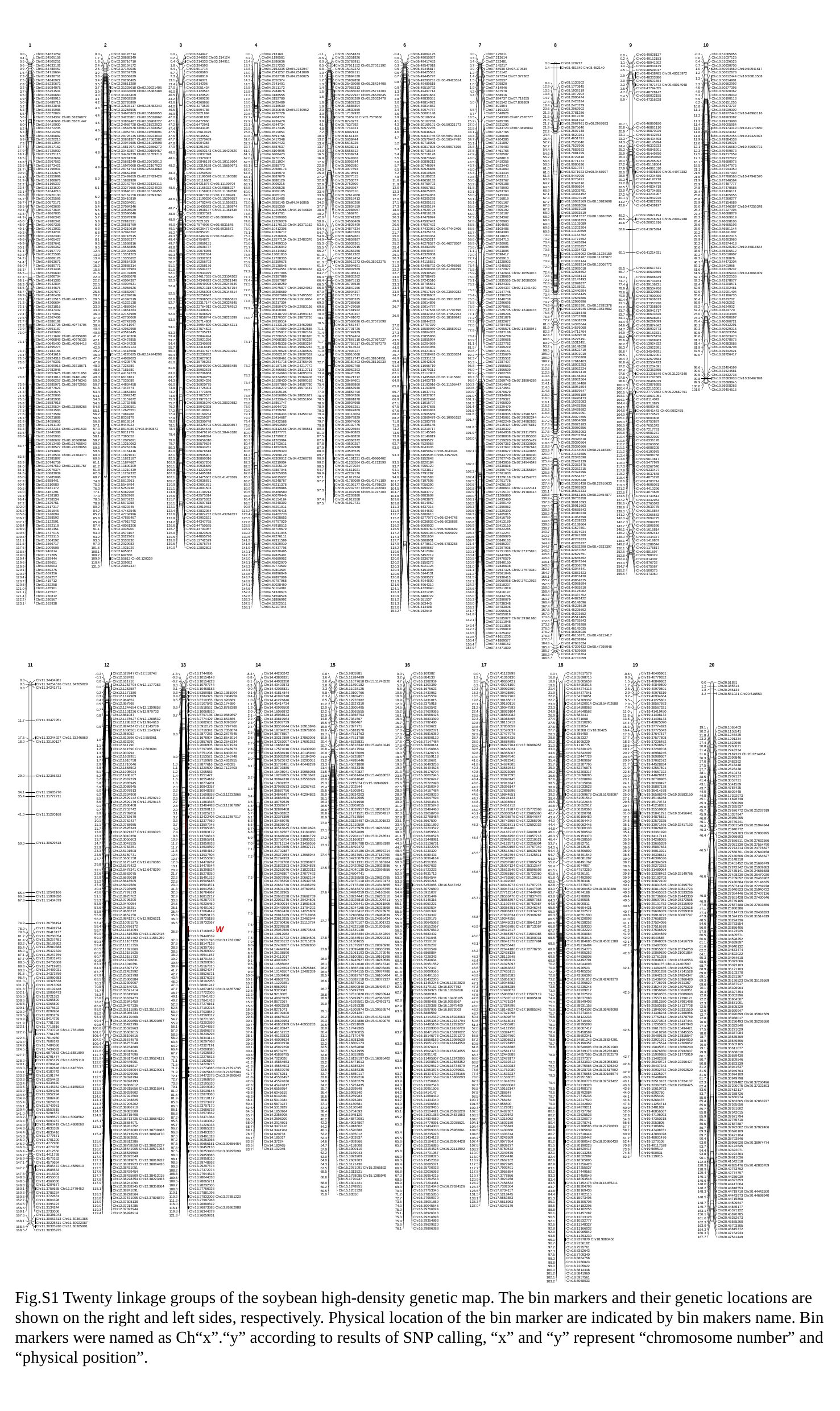

Fig.S1 Twenty linkage groups of the soybean high-density genetic map. The bin markers and their genetic locations are shown on the right and left sides, respectively. Physical location of the bin marker are indicated by bin makers name. Bin markers were named as Ch“x”.“y” according to results of SNP calling, “x” and “y” represent “chromosome number” and “physical position”.
